# Pseudomyxoma peritonei – a revisit: report of 2 cases and literature review

**DOI:** 10.1186/1477-7819-4-60

**Published:** 2006-09-01

**Authors:** Chunyanca Li, Rani Kanthan, SC Kanthan

**Affiliations:** 1 Department of Pathology, Royal University Hospital, 103, Hospital Drive, Saskatoon, Saskatchewan, SK S7N OW8, Canada; 2 Department of Surgery, Royal University Hospital, 103, Hospital Drive, Saskatoon, Saskatchewan, SK S7N OW8, Canada

## Abstract

**Background:**

Pseudomyxoma peritonei (PMP) is a rare, chronic, relapsing, diagnostically challenging and poorly understood disease characterized by disseminated mucinous ascites and peritoneal implants.

**Case presentation:**

We report two cases of PMP that represent the two biological variants of **d**isseminated **p**eritoneal **a**deno**m**ucinosis (**DPAM**) – the benign variant and the **p**eritoneal **m**ucinous **c**arcinom**a**tosis (**PMCA**) – the malignant variant, both of which were characterized by multiple relapses and progression of the disease despite aggressive management.

**Conclusion:**

Even with a better understanding and recent advances in the management of these cases, PMP remains an enigmatic disease with a protracted clinical course characterized by multiple recurrences despite surgery and/or chemotherapy. Recognition of PMP as a delayed consequence years later should alert all surgeons to be extremely vigilant when treating mucinous neoplasms of the appendix, with special care being directed towards adequate excision and thorough debridement at the initial diagnosis.

## Background

Pseudomyxoma peritonei (PMP) first described by Werth [1] is an uncommon and poorly understood disease characterized by abundant extracellular mucin in the peritoneum. The "myxomatous" appearance is attributed to the associated fibroblastic and vascular proliferation that is probably incited by the mucin. This results in multifocal peritoneal, serosal and ommental implants admixed with copious amounts of mucin accumulation within the abdomen and pelvis resulting in the belly full of jelly – "the jelly belly" [2]. PMP is a broad descriptive term embracing a wide spectrum of biological behavior of neoplasms from the benign to the borderline to the frankly malignant lesion. Other terminologies to reflect this spectrum of biological behavior ranges from **d**isseminated **p**eritoneal **a**deno**m**ucinosis (**DPAM**) – the benign variant to **p**eritoneal **m**ucinous **c**arcinom**a**tosis (**PMCA**) – the malignant variant [3]. A definitive diagnosis of PMP requires the presence of a) mucinous neoplastic cells/epithelium, and b) mucinous ascites – diffuse intraabdominal mucin. Some authors also require the presence of diffuse mucinous implants for this diagnosis. Viable epithelial glandular cells must be identified within the mucin pools by histological analysis to diagnose PMP. Cases without epithelium are regarded as mucinous ascites.

Pseudomyxoma peritonei (PMP) is more commonly seen in women [4] who usually present with increasing abdominal girth and this tends to be related to an underlying ovarian lesions which are usually mucinous tumors that can be associated with **a **teratoma. Though uncommon in men, these cases are virtually all associated with a lesion in the appendix [5]. Other possible primary sites include colorectum, gallbladder, pancreas, urachus, urinary bladder, breast and lung [6], but these are uncommon. These primary sites are usually associated with the malignant variant-PMCA of PMP. PMP can occur years (range from 5–35 years) later after the initial presentation of an appendiceal event [7] and, therefore, accurate diagnosis prior to surgery is often delayed and inaccurate. The disease may be localized in the right lower quadrant initially and then become more generalized with mucinous peritoneal, serosal and ommental implants. There is increasing recognition that the two variants DPAM and PMCA are different with the DPAM remaining localized to the abdomen without metastatic behavior and the PCMA behaving like a mucinous (colloid) carcinoma with metastatic and invasive potential. 10% of patients die of PMP within 5.5 years of their initial presentation while others experience recurrent and/or residual disease. Advanced abdominal disease leading to intestinal obstruction accounts for majority of the patients' morbidity and mortality. We report two cases of delayed PMP in men associated with appendiceal lesions with a long latent period in one case. Although both underwent surgery and one had adjuvant chemotherapy, a protracted clinical course with chronicity and relapse of the disease was a common feature.

## Case presentation

**Case 1 **This is a 42 years old male with a history of a ruptured appendiceal mucinous neoplasm that was removed 23 years ago. He was asymptomatic until the fall of 1999 when he started complaining of recurrent small bowel obstruction and weight loss. He had undergone multiple investigations and was thought to have underlying Crohn's disease. A computed tomographic (CT) scan of his abdomen in January 2000 demonstrated a large abdominal mass. A laparoscopy was performed for tissue diagnosis of a possible lymphoma. However, at the time of laparoscopy, a large gelatinous mass was found and the diagnosis of PMP was confirmed by histopathology. Debulking surgery with small bowel resections was performed following this diagnosis at the Royal University Hospital of Saskatchewan. Since then he remained well and was reviewed regularly in the outpatient clinic. Four years later (2003), the patient began experiencing obstructive symptoms again due to recurrent disease and he had a further extensive debulking surgery in January 2004. This was followed a year later by another cytoreductive surgery with small bowel resection in 2005. He is currently doing well at a 3 months post-operative follow-up visit.

### Pathology findings

**Figure 1:**
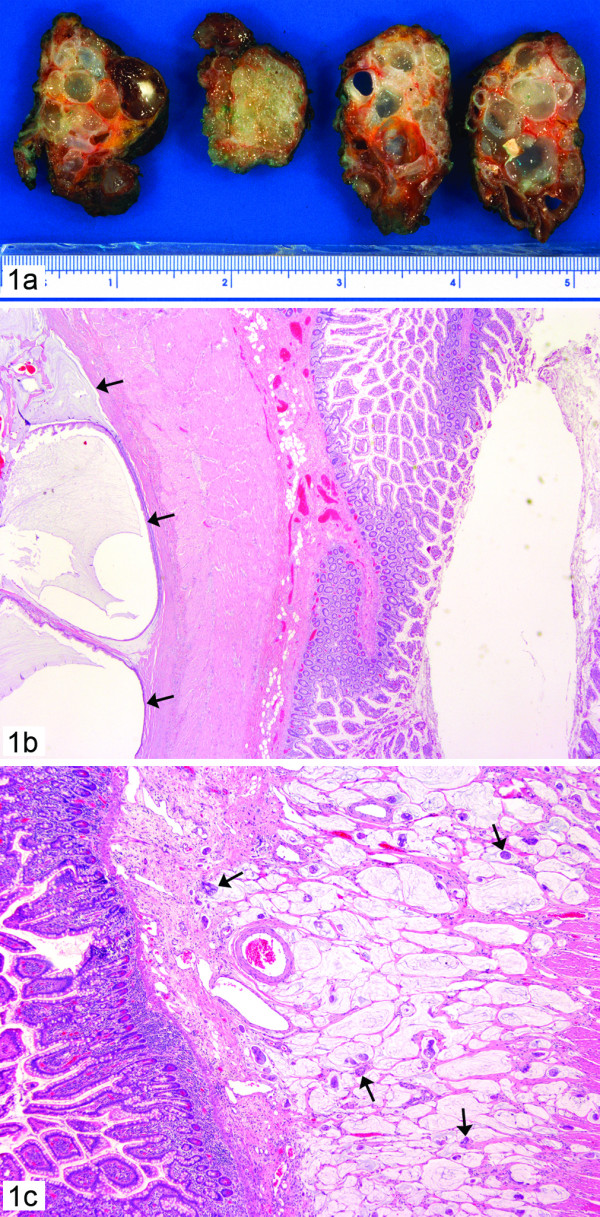
Case 1: 1a. Pseudomyxoma peritonei with multilobulated polypoid gelatinous masses, 1b. Subserosal cystic structures filled with mucin and lined by single layer of bland epithelium (arrow) in the small bowel. (hematoxylin-eosin, × 10); 1c. Numerous cell clusters floating in the mucin pools and infiltrating the submucosa of small bowel (arrow) (haematoxylin-eosin, × 20)- PMCA variant of PMP

The specimens from 2000 consisted of a portion of small bowel with several gelatinous, "mucinous" masses attached to the peritoneal surface of the serosa of the bowel wall accompanied by the presence of lobulated polypoid gelatinous tissue (Figure 1a). The specimens from 2004 and 2005 had a similar gross appearance. Histologically, however, the tumor showed significant progression over the years from a predominantly hypocellular, benign looking single layer of bland epithelium in extracellular pools of mucin in year 2000 (Figure 1b) to a mild increase in epithelial proliferation in 2004 to a hypercellular lesion associated with cytological atypia and numerous free "cell clusters" floating in the extracellular mucin pools and infiltrating the submucosa of the small bowel (Figure 1c) representing the PMCA variant with a documented past history of ruptured appendiceal mucinous neoplasm 23 years ago with no material available for current pathological review.

**Figure 2:**
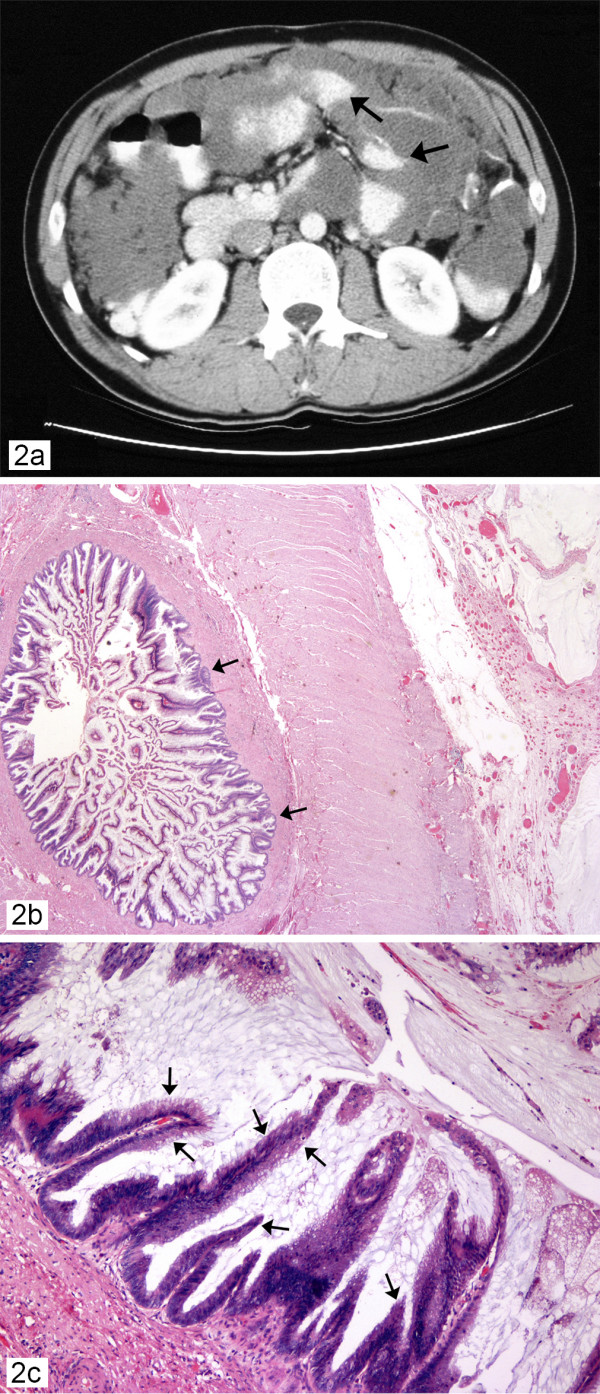
Case 2 ; 2a. Computed tomographic scan of abdomen showing pseudomyxoma peritonei with multiple peritoneal masses (arrow) with "scalloping effect" seen. 2b. Prominent papillary architectures (arrow) (haematoxylin-eosin, × 10); 2c. Papillary architectures with mild to moderate nuclear atypia (arrow) (hematoxylin-eosin, × 20) with no invasion-representing a case of the DPAM variant of PMP.

**Case 2 **This was a 63-year-old male who presented with fatigue and dyspnea in the spring of 1995. He was investigated for his anemia and found by ultrasound to have multiple cystic structures within the peritoneal cavity. The CT scan confirmed the presence of multiple peritoneal masses associated with a large amount of intra-abdominal ascites (Figure 2a). At laparoscopy the abdominal cavity was filled with mucin. Biopsy and histopathological examination of the peritoneal mass confirmed the presence of PMP. Cytoreductive surgery inclusive of a right hemicolectomy was performed, followed by intra-peritoneal chemotherapy. The patient had been doing well for 4 years. Then, he complained of increased abdominal girth and coughing, and a CT scan showed recurrence of extensive amount of multiloculated material throughout the abdomen and pelvis. Therefore, the second debulking surgery was performed on September 1999. The patient made an uneventful recovery and was discharged in a stable condition. In June 2002, he had the third debulking surgery for recurrent disease. In February 2003 he died from a myocardial infarction – an unrelated cause.

### Pathology findings

The gross appearance of the PMP was similar to the previously described case. A diagnosis of PMP originating from a mucinous neoplasm of low malignant potential (M-LMP) originating in the appendix from the hemicolectomy specimen of 1995 was confirmed microscopically. The lesion had prominent papillary architecture with mild to moderate nuclear atypia (Figure 2b, 2c). Foci of pseudomyxoma extraperitonei were also observed in sections of the skin. The tumor cells from the next two debulking specimens showed similar histological features as the first one with no obvious progression from the original state of the bland epithelium – no significant increase in the degree of atypia, cellular proliferation, increased nuclear hyperchromasia, abnormal mitoses or the presence of signet ring cells representing a case of DPAM variant of PMP arising from a borderline or low malignant potential (LMP) mucinous neoplasm of the appendix.

## Discussion

Pseudomyxoma peritonei is an indolent disease and preferentially affects women with an average age of 53 years [4]. It is traditionally believed that most cases of PMP originate from ovarian tumors. This belief is challenged recently by increased usage of immunohistochemical stains and molecular genetic studies, which showed a large proportion of these tumors to be secondary to appendiceal tumors in both men and women [5,8-10]. Although we present 2 cases of male PMP, the fact remains that more women than men suffer from this condition according to the published literature [4].

As symptoms remain non-specific the disease presents a great diagnostic challenge to clinicians. Clinical presentation is late and patients usually experience a long course of health deterioration before an accurate diagnosis is made. Due to its indolent nature, advanced stages of the disease with generalized peritoneal tumor implants, fistula formation and adhesions are common. In this advanced stage, abdominal symptoms caused by partial or complete bowel obstruction are the main complaints.

A precise diagnosis is difficult due to the lack of specific symptoms in the early stage of the disease. Routine laboratory studies are seldom helpful in making this diagnosis. An accurate preoperative diagnosis of pseudomyxoma peritonei can be aided by radiological imaging with computed tomography if the characteristic "scalloping effect" on the surface of the visceral organs resulting from compression by the viscous mucinous secretions and the organizing fibrosis is seen as observed in figure 2a. [1]. The role of magnetic resonance imaging as a diagnostic tool is unclear. In the majority of cases, it is often an unexpected finding at explorative laparoscopy, which remains the main diagnostic tool with the final diagnosis being confirmed by histopathology.

Mucinous neoplasms of the appendix are uncommon entities associated with a variety of underlying pathological processes ranging from simple appendiceal mucoceles to mucinous hyperplasia (hyperplastic polyp), serrated adenoma, mucinous adenoma/cystadenoma, mucinous neoplasm of uncertain malignant potential, mucinous neoplasm of low malignant potential and mucinous cystadenocarcinoma. [11] The exact relationship of these lesions to PMP is still not clear though many cases of PMP, predominantly the DPAM variant, are associated with mucinous neoplasms of the appendix with low malignant potential (M-LMP) as in case 2. The PMCA variant of PMP seems to have a higher association with mucinous adenocarcinomas of the appendix and other rarer gastrointestinal sites. The pathological features of a borderline or low malignant potential appendiceal mucinous neoplasm consists of glands lined by mucinous epithelium with small basally located nuclei and inconspicuous nucleoli in a mucinous background as in a mucinous adenoma. The key associated finding is the presence of epithelium outside the appendix in association with the mucin and the peritoneal implants as observed in Case 2. A cystic ovarian tumor should always be excluded in women with an appendiceal mucinous neoplasm. The correct diagnosis of appendiceal mucinous neoplasm of low malignant potential (M-LMP) is almost never made pre-operatively and is suspected intraoperatively in about a third of the cases. These cases often have a long protracted clinical course with death occurring decades after the initial diagnosis. Death may also occur in the interim period due to unrelated causes as in our indexed Case 2. Death directly related to PMP in these cases is due to extensive peritoneal fibrosis with bowel obstruction and not as result of lymph node, liver or lung metastases. Appendiceal mucinous cystadenocarcinoma however, should demonstrate overt cytoarchitectural features of carcinoma including cell clusters with significant cytological atypia, stratification, papillary formation with invasion of the bowel wall. These cases are associated with an aggressive clinical course often with liver, lung and lymph node metastatic disease. [11].

PMP on the other hand can be associated with a mucinous neoplasm either in the ovary or in the appendix that demonstrate fairly bland well differentiated mucinous epithelium with minimal nuclear features of malignancy, which further confirms that the origins and nature of the parent tumors may be variable and that the cytological features are not always concordant with those of PMP further adding difficulty in determining its biological behavior and its subtype. Thus the natural history of this disease remains largely poorly defined.

Recent studies reveal that PMP is a neoplastic disease of MUC2-expressing goblet cells. Mucinous tumors of the appendix also express MUC2, which supports an appendiceal rather than an ovarian origin for PMP. This helps to distinguish PMP secondary involvement of the ovary by PMP from a primary appendiceal mucinous tumor with peritoneal implants [12]. The exact origin and mechanism of this abundant mucin production has been recently extensively studied. It is believed that PMP is characterized by the production of MUC2, a gel forming mucin that forms strong bonds with the surrounding stroma and is also believed to have tumor suppressor activities [12]. The extracellular accumulation of mucin is attributed to an alteration in cell polarity resulting in the glycoproteins being secreted predominantly in the stroma facing surfaces of the tumor cells in contrast to adenocarcinomas wherein the glycoproteins are secreted either into the luminal surface or dispersed within the cytoplasm [13].

The evolution of treatment strategies of PMP still remains debated though the current mainstay of the treatment remains surgical extirpation of the lesion. Repeated cytoreductive surgical debulking procedures including resections of the tumor implants, omentum and obstructive bowel are common due to recurrence of the disease. Intra-peritoneal chemotherapy (5FU, Mitomycin C) has minimal benefit with reduction in the number of foci of atypical epithelium and or atrophy and degeneration of the atypical neoplastic epithelium. Based on the Sugarbaker peritonectomy procedure [14] a study by Draco *et al *showed that cytoreductive surgery with intraperitoneal hyperthermic perfusion permitted complete tumor removal, and this study confirmed the efficacy of this combined treatment in terms of improved long-term survival and better local control of the disease [15]. Indeed, the current strategy of treatment includes cytoreductive surgery combined with intraoperative hyperthermic intraperitoneal chemotherapy (mitomycin at 42 degrees C). The aim is to avoid entrapment of tumor cells at operative sites and to destroy small residual mucinous tumor nodules. This is a combined treatment with a high toxicity, particularly surgically related [16], but is associated with long term survival compared to the traditional historical treatment [17]. As endorsed by Sugarbaker, this new combined treatment should be regarded as the standard of care for epithelial appendiceal neoplasms and pseudomyxoma peritonei syndrome [18] which is best administered in an established peritoneal carcinomatosis treatment centre.

Prognosis in this disease is closely related to the bulk of the disease as evaluated by the tumor site, preoperative tumor volume and completeness of tumor removal by cytoreductive surgery and the microscopic degree of differentiation of the neoplastic epithelium as evaluated by the histopathological examination [17-20]. In this context, PMP patients with pre-operative elevated tumor markers such as CEA (carcino-embryonic antigen) and CA 19-9 are at increased risk of developing recurrent disease despite aggressive therapy. Likewise, PMP patients with normal levels of these tumor markers have an overall improved prognosis [21,22].

## Conclusion

We have described two male patients who developed pseudomyxoma peritonei arising from appendiceal tumors with a latent period of 23 years in one case. They both were diagnostically challenging and were accurately recognized at surgery for bowel obstruction and confirmed by histopathology. They represent the two variants of PMP – the malignant variant of PMCA and the benign DPAM with well-differentiated tumor who despite tumor recurrences died of an unrelated event. The former is treated aggressively with cytoreductive surgery and chemotherapy while the latter is principally managed by surgical debulking procedures. Awareness of this indolent and rare condition is an important prerequisite for early diagnosis and appropriate management. Further, clinical awareness and recognition of PMP as a potential delayed consequence years later to an appendiceal lesion should alert all surgeons to be extremely vigilant while treating mucinous neoplasms of the appendix, with special care being directed towards adequate excision and thorough debridement at the initial diagnosis.

## Competing interests

The author(s) declare that they have no competing interests.

## Authors' contributions

**CL **is the pathology resident who prepared the manuscript

**RK **is the consultant pathologist who handled these cases, supervised the resident and edited the manuscript for its scientific content

**SCK **is the senior author and the surgeon in charge who helped in preparation of manuscript and edited it for its scientific content.

All authors read and approved the final manuscript
